# A Self-Optimizing Scheme for Energy Balanced Routing in Wireless Sensor Networks Using SensorAnt

**DOI:** 10.3390/s120811307

**Published:** 2012-08-15

**Authors:** Ahmed M. Shamsan Saleh, Borhanuddin Mohd Ali, Mohd Fadlee A. Rasid, Alyani Ismail

**Affiliations:** Department of Computer and Communication Systems Engineering, Universiti Putra Malaysia, 43400 UPM Serdang, Selangor, Malaysia; E-Mails: borhan@eng.upm.edu.my (B.M.A.); fadlee@eng.upm.edu.my (M.F.A.R.); alyani@eng.upm.edu.my (A.I.)

**Keywords:** energy balancing, energy consumption, ant colony, battery lifetime, WSNs

## Abstract

Planning of energy-efficient protocols is critical for Wireless Sensor Networks (WSNs) because of the constraints on the sensor nodes' energy. The routing protocol should be able to provide uniform power dissipation during transmission to the sink node. In this paper, we present a self-optimization scheme for WSNs which is able to utilize and optimize the sensor nodes' resources, especially the batteries, to achieve balanced energy consumption across all sensor nodes. This method is based on the Ant Colony Optimization (ACO) metaheuristic which is adopted to enhance the paths with the best quality function. The assessment of this function depends on multi-criteria metrics such as the minimum residual battery power, hop count and average energy of both route and network. This method also distributes the traffic load of sensor nodes throughout the WSN leading to reduced energy usage, extended network life time and reduced packet loss. Simulation results show that our scheme performs much better than the Energy Efficient Ant-Based Routing (EEABR) in terms of energy consumption, balancing and efficiency.

## 1. Introduction

Even though Sensor Nodes (SNs) may link directly to the processing sinks through local area networks [1], extending these SNs to gather data and transmit them to a central sink is more useful because future applications may demand hundreds or thousands of SNs to be deployed, which are often used in remote and inaccessible regions. A single SN may not only have a sensing capability but also processing, communications and memory units [2], which means that a SN can perform data collection, in-network processing and fusion of its own and other SNs' information.

**Figure 1.**
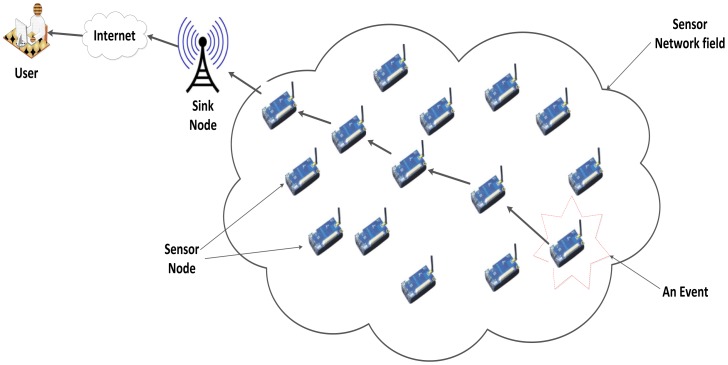
Typical wireless sensor network.

When many SNs join to observe an enormous physical environment thus they form what is termed a Wireless Sensor Network (WSN). WSNs have many applications such as environmental monitoring, health care, security, tracking events and industrial automation [1]. Figure 1 shows a typical wireless sensor network.

Data routing is a very critical process in WSNs due to the communications range constraints [3]. Therefore, it is preferable to forward packets from the collection area to the sink or base station by multiple hops in order to reduce the SNs' energy consumption. Consequently, while designing a routing protocol, energy awareness should be taken into consideration such that the battery power is utilized efficiently in order to prolong the lifetime of both nodes and network. Moreover, distributing traffic load across multi-paths balances their energy usage and optimizes WSN throughput. By avoiding frequent use of some nodes it prevents the partitioning of WSN which shortens its life span. Hence this proposed method ensures that the SNs will drain their batteries evenly across the network.

There are many challenges and constraints between WSNs and other distributed networks despite their similarities. These constraints affect the design of WSN; this leads to the need to develop protocols that consider these constraints in order to work in WSN environments. The restriction is most often associated with the design of SNs with limited battery power [3,4]. Typically, they are equipped with batteries that have to be replaced or replenished, if they are exhausted. In most cases, it is very difficult to do this, especially in harsh or remote areas, therefore, it is very important to manage the energy consumption in order to conserve energy and ensure longer survival. Therefore, SNs should cooperate and adapt to failures and changes in the phenomena without any external intervention.

Routing protocols are an active research topic in sensor networks, and many such techniques have been published [5]. Some of these routing results from the adaptation of the conventional routing methods which are not appropriate for wireless networks because of resource constraints and frequently changing topology. Due to these challenges new techniques must be adopted to cope with them. Biological inspiration (BI)-based techniques [6], define a new set of rules that can be adapted for WSNs in terms of gathering the food and communications among the species such as ants, bees and birds. The basic idea is that these BIs have to be adjusted to develop new protocols for optimizing and managing sensor networks. Some of these schemes are inspired by the foraging activities of ants. The main reason is that these insects act as a collective unit to resolve routing challenges. They go around to discover and establish routes used by individual ants to efficiently move forward and backward from the nest toward the target food [7]. These routes arise from the cooperative connections of the numerous ant agents during the routes sampling to notify the others about their properties indirectly. The searching behavior of ant agents proves to be adaptive to ambient changes with more scalability.

The majority of routing protocols for WSNs aim at decreasing the energy usage of sensor nodes by either explicitly taking energy into account during the route selection or by optimizing some metrics. Even so, in some cases these mechanisms may cause the sudden death of critical nodes that lead an entire network to malfunction. This is because critical nodes experiencing heavy traffic load deplete their energy fast and die out, whilst nodes in sparse regions in terms of data traffic continue to enjoy high energy levels. Some solutions have been introduced for these problems but they still suffer from the quick draining of the critical sensor nodes and do not utilize the other sensors that still have near full power capacity inside the network; this shortens the life time of network since it is determined by the energy of the sensor nodes.

Thus the motivation of this study was to propose a decentralized energy balancing method that is generic and applicable to most of WSN applications that require reduction of energy consumption and this will therefore extend a network's life time. This is achieved by considering the energy of the sensor nodes as the most critical factor to prevent fast energy depletion. In this method the traffic load are regularly and slowly distributed over all the sensor nodes during routing which will minimize their energy usage averaged over the network; the sensors die almost all at the same time which will in turn optimize the overall network life time.

The contributions of the paper are: firstly, in developing a routing algorithm whose main consideration is to balance the energy consumption in order to maximize the sensor network lifetime. Secondly, combining the simplicity of BI with the efficiency of diffusion scheme in routing to introduce a SensorAnt mechanism based on Ant Colony Optimization (ACO), which uses two quality assessment functions for hop and path to select the best next hop and update the routing tables of nodes efficiently. Finally, presenting a new hop function assessment based on multi-criteria metrics such as the minimum residual battery power, hop count and average energy of both route and network, to contribute to the path function choosing the optimal route to the destination.

We compared their performance and show that it gives a better performance compared with the Energy Efficient Ant-Based Routing algorithm (EEABR) [8]. The EEABR is a well-known WSN algorithm that essentially uses an ACO-based system. The authors of the EEABR protocol compare it with another two algorithms; the basic ant based routing (BABR) and the improved ant based routing (IABR) and both use the ACO metaheuristic. In this way the EEABR is considered a good benchmark in most of WSN routing protocols based on ACO. Simulation results show that our proposed model reduces energy consumption in WSNs and optimizes the number of packet drops efficiently which extends the lifespan of the network.

The rest of this paper organized as follows: Section 2 reviews some of the related work, Section 3 describes in detail our proposed method, while Section 4 presents the performance evaluation of our method. The last section summarizes our work.

## 2. Related Works

In this section, a background review of the energy awareness routing protocols in wireless sensor networks are presented. The majority of routing protocols for WSNs aim at decreasing the energy usage of sensor nodes by either explicitly taking energy into account during the route selection or by optimizing some metrics, such as minimizing the energy consumed per packet, reducing the cost/packet ratio, or minimizing the high energy depletion of any one sensor.

Approaches which minimize the energy consumed per packet in many cases lead to poor routes in that some sensor nodes may be unnecessarily overloaded and thus rapidly drain their batteries. One way to solve this problem is to maintain a balanced residual energy for all sensor nodes in the network. This is because the normal operation of the network may cease when a few nodes run out of energy, even if the majority of the nodes still have a near-full battery capacity. Moreover, choosing the lowest energy cost paths may not always extend the lifetime of network because some of the sensor nodes over those paths may have limited residual energy, and hence, will deplete their energy very fast. As such, distributing the traffic load and energy consumption fairly across the sensor nodes is a more desirable strategy for routing protocols.

Many energy efficiency MAC and routing protocols have been presented to reduce energy usage in WSNs using sleep mode to turn off some parts of SN, to saving energy, during idle mode. In [9], the authors presented a dynamic awakening method, that makes sleep time longer for SNs by estimating the idle phase for each node during sensing and transmission mode. Hence, the SNs within the desired object range switch to active mode on time and serve like sensing candidates. Their dynamic scheme improves the energy efficiency and reduced the total consuming energy in the sensor network. The authors in [10], proposed a wake-up MAC scheduling which switches the nodes from active to sleeping mode whenever required. In [11,12], new network layer energy efficient routing strategies are proposed, where the sleep states of some nodes were considered as well.

### 2.1. Energy-Aware ACO-Based Routing in WSNs

Ant Colony Optimization (ACO) is possibly the best studied field in swarm intelligence (SI) algorithms. Usually, SI is the basis of studying the collective behavior of distributed, self-configuring principles such as ant colonies [13]. The main rules of the ant systems are generally performed locally from a population of ant agents interacting with each other and their environment. These agents are capable of solving complex tasks with simple resources. There is only indirect communication between agents via their surroundings adjustment, for example, a pheromone trail used to forage efficiently. Ants first work randomly throughout the foraging process, then they follows the same path to the nest which is indicated by the pheromone. During the return journey more pheromone trace is deposited to show the direction of destination by tracking this trail on the shortest route. Ants communicate via changes in the ambient-pheromone trace, this process is called stigmergy. The details of the ACO algorithm can be found in [14].

In [8], the Energy Efficient Ant-Based Routing (EEABR) was designed to extend the lifetime of WSNs by decreasing the communication overhead in the discovery phase. This is attained by way of two factors—energy and hop count when updating the pheromone. In addition, they use a fixed ant size to construct energy efficient routes. Ants are generated proactively in EEABR at regular intervals and unicasted to the next hop SNs that is selected by a probabilistic rule. The EEABR uses the same probability and visibility function for taking the decision to select the next hop as that in the many ant-based system with the only difference being the metrics evaluation. Additionally, updating the pheromone table in EEABR follows the same method used in the others; our SensorAnt protocol proposes a new method for calculating the probability and the method of updating using two functions assessment with the multi-criteria decision which, to the best of our knowledge, has not been reported prior to this. Furthermore, the EEABR is a weak in terms of scalability and reliability. Despite these differences between EEABR and SensorAnt both share some significant characteristics such as using the distributed mechanism, the traffic generation is event driven, both are address-centric rather than data-centric and the two schemes operate in a flat topology. Moreover, most of the Energy Efficient routing protocols based on ACO take the path decision from the cost energy based source routing rather than next hop that concentrates on the residual energy of the sensor itself. EEABR and SensorAnt exploit this mechanism for evaluating the routes. Hence, these similarities which are considered more realistic in most of the WSN applications motivate us to compare our proposed algorithm with EEABR in order to prove the superior effectiveness of our method.

In [15], the authors first proposed a routing based-on centralizing offline ant colony system to solve the Steiner tree problem. Later they presented a distributed online-based method for data centric routing in sensor networks. The WSN is presented as a weighted graph where a weight-cost of an edge is the Euclidean distance of the connected SNs. The object is then to obtain a Steiner tree of minimum cost when forwarding packets to the sink. The authors built their method using a unique base-station. This scheme introduces a good design for multicasting trees in WSNs. Moreover, the authors suggest that the destination creates the ants-backward only after the entire ants-forwarded are received, and the same condition can be applied to the ants-forwarded which will not be created from the sensors until all the ants-backward are back. So this method is difficult to achieve in reality, because some ants may be lost during the transmission process, which is normal in unreliable sensor networks.

Many-to-One Improved Ant Routing (MO-IAR) in [16], proposed an ant-based protocol that is designed for upstream routing many-to-one sensing packets. The routing algorithm is linked with a congestion control scheme that helps to alleviate the collisions. The protocol is divided into two stages; during the first stage, it finds the shortest route between any SN and the destination while in the second stage it exploits the shortest route to prevent the congestion and minimize packet loss. The protocol assumes that the location of each SN, the target, and their neighbors are known. The disadvantage of this method is that the selection of the best route is based only on the distance, and there is no consideration of the residual battery power.

The authors in [17] proposed an Ant-aggregation method for sensor networks. Their method used multi-hop connections, including in-network processing resulting in significant improvement in the lifespan of the WSN by decreasing battery power demands. Their method constructs the trees that satisfy the smallest cost accumulation. Furthermore, the mission of the ants is to find the shortest route to sink or to the closest aggregation node.

An efficient routing for large cluster based WSNs has been presented in [18], by applying a pair of routing levels. Firstly, it uses the intra cluster where the SNs inside the cluster transmit their packets immediately to the head. Next, they use ant-based systems for the inter-cluster among the cluster heads to discover the path to the sink node. Their method managed to reduce the latency of the algorithm and provides a more effective operation.

### 2.2. Energy-Aware Optimization-Based Routing in WSNs

A prominent energy-efficient algorithm for clustered networks is the Low Energy Adaptive Clustering Hierarchy (LEACH) [19]. LEACH uses data aggregation techniques and a cluster head selected in a random way. It is a routing model based on hierarchical topology and energy efficiency. The main drawback in LEACH is that it is unsuitable for time critical applications. In addition, LEACH suffers from scalability problems due to the assumption that every sensor can transmit directly to the sink. The authors in [20], proposed the Routing based on Energy-Temperature Transformation (RETT-gen), which is a modification of the LEACH protocol, taking into consideration the residual energies of the sensor nodes in routing decisions. They disseminate equally the energy load over all SNs in the network, thereby extending the network lifetime. However, the persistent problem in this protocol and other clustering protocols is that the cluster formation process as well as the probabilistic cluster-head selection incurs high overheads and complexity.

The power-efficient gathering in sensor information systems (PEGASIS) [21], introduces a new factor delay-energy, which concurrently decreases delay and energy cost. PEGASIS is similar to LEACH but uses lower energy per round. It creates a chain such that if a sensor has data to send to the sink, it goes through the leader sensor, which is chosen randomly every round. However, one of the limiting factors of PEGASIS is that every sensor has to aggregate data such that packet transmission is minimized. Moreover, the protocol requires the global information of the network and uses greedy method before the chain formation can be done, which requires data packet traversal through many sensor nodes.

In [22], a threshold-sensitive energy efficient sensor network protocol (TEEN) is proposed. TEEN is an adaptive protocol for frequently changing network conditions in clustered WSNs. The problem of this protocol is the high incurred overhead during cluster formation. Additionally, the protocol cannot recognize dead sensor nodes.

The authors in [23] proposed the self organized routing (SOR) protocol, where nodes conserve energy by employing other nodes as mediators to increase the survival time of the network. They introduced payment schemes for sensor nodes that act as mediators. Payment received by a node depends on whether it transmits its own packet or acts as a mediator for others. The protocol is energy efficient due to the self interest orientation of the nodes, using local information to make informed decisions.

The authors in [24], proposed Direct Diffusion (DD) for data centric based routing protocols. The protocol uses the data address rather than the IP address. Here, the user sends a query to the sensor network for specific events and those nodes that sensed the required event start to send their data. However, the DD scheme does not consider the residual energy of nodes in the flooding phase and continuously broadcasts the request of interest, which causes high energy dissipation due to transmission and reception.

The authors in [25], proposed the so-called Minimum Energy Communication Network (MECN) for *ad hoc* network scenarios. The protocol also works well with wireless sensor networks. The improved version of MECN, which is known as Small-MECN (SMECN) shows a better performance, offering an energy efficient sub-network. The sub-network includes all nodes from the original network and a subset of the edges.

The work in [26], proposed a distributed energy balanced routing (DEBR) protocol, which decentralizes the data traffic in the network in a way that prolongs the lifetime of the network. The algorithm makes a tradeoff between packet delay and energy. Despite the applicability of the algorithm in many WSN scenarios, it assumes that each SN is within the direct communication range of the sink, which is not true for multi-hop routing.

## 3. The Proposed SensorAnt Protocol

As has been highlighted before, WSNs have strict energy requirements. Hence, the routing protocols should take into consideration these constraints to develop algorithms that have the capability to distribute the load traffic, in order to avoid the extra loading of nodes at the same route. As a result, the life time of the sensor nodes can be extended, while the energy balancing and energy efficiency will be satisfied at the same time.

### 3.1. The Network Model

A WSN can be designed as a connected graph *G(V, E)*, where *V* is the set of sensor nodes and *E* represents the set of communications links. Let 1 represents two sensor nodes which are directly connected while 0 represents two unconnected sensor nodes. Given a sensor node *υ* ∈ V with transmission range *R*, if *υ* is transmitting, all nodes within the carrier sensing of *v* will not be able to access the medium when the channel is busy. The transmitted packets from *υ* can follow one of the possible paths in the graph *G(V, E)* that connects *υ* to the sink node. In the proposed model, a node can transmit data packets to any neighbor within a range. All sensor nodes are assumed static and are deployed randomly and with a finite battery power. All the sensor nodes have equal initial energy.

### 3.2. The Energy Model

Energy consumption in WSN is mainly attributed to the radio system being in active mode. In fact, the energy consumed in the sleep and idle modes are very small compared to the transmit and receive modes. Let sensor node *x* have *N* bits of packets to transmit or receive during active mode (per unit time). Accordingly, the total energy consumption for *x* can be calculated as:

(1) $$\text{ToEnC}(x)=EnC^{Tx}(x)+EnC^{Rx}(x)$$

where the *EnC^Tx^*(*x*) and *EnC^Rx^*(*x*) are the circuit energy consumption for the node *x* during the transmission and receiving states respectively. The energy consumed due to transmission *EnC^Tx^*(*x*) is given by:

(2) $$EnC^{Tx}(x)=EnC^{\text{elecT}}+EnC^{\text{amp}}$$

where *EnC^elecT^* is the electronic circuit energy consumed due to the transmitter, which is calculated as:

(3) $$EnC^{\text{elecT}}=EnC^{mx}+EnC^{\text{Sny}}+EnC^{\text{filt}}+EnC^{\text{dac}}$$

where *EnC^mx^*, *EnC^Sny^, EnC^filt^ and EnC^dac^* are the energy consumption of the mixer, frequency synthesizer, filter and digital to analog converter respectively, and *EnC^amp^*, the energy consumed by the power amplifier.

In the same manner, the energy consumption in the receiving state *EnC^Rx^* (*x*) is given by:

(4) $$EnC^{Rx}(x)=EnC^{\text{elecR}}$$

where *EnC^elecR^* is the electronic circuit energy consumption at receiver and calculated as:

(5) $$EnC^{\text{elecR}}=EnC^{mx}+EnC^{\text{Sny}}+EnC^{\text{filt}}+EnC^{\text{lna}}+EnC^{\text{ifa}}+EnC^{\text{adc}}$$

where *EnC^lna^, EnC^ifa^* and *EnC^adc^* are the energy consumption of the low noise amplifier, the intermediate frequency amplifier and the analog to digital converter respectively.

Based on Equations (1), (2) and (4), the energy consumption per packet bits *EnC_pkt_* (*x*) is given by:

(6) $$EnC_{\text{Pkt}}(x)=\text{ToEnC}(x)/N$$

### 3.3. The Lifetime Model

The network lifetime in a sensor network depends on many factors, including the energy model, frequency coverage, message size, and aggregation schemes [27]. Therefore, WSNs should have to select the suitable elements that allow the protocol design and deployment to elongate sensor network lifetime. Accordingly, this paper measured the lifetime based on the energy consumption model. Hence, the lifetime of the network is declared as the time spent from deployment until the first sensor or part of sensors becomes unable to transmit packets to its neighbors because it has drained its energy.

Therefore, the lifetime of sensor node *x* based on Equation (6) is given by:

(7) $$LT(X)=\frac{E_{\text{init}}}{EnC_{\text{Pkt}}(x)}$$

where *E_init_* is the initial energy of the sensor node.

This means that the lifetime of the greediest node in the network must be maximized in order to improve the network lifetime since this node has a direct impact upon the whole WSN life according to the declaration of the lifetime. This problem can be represented by the following minimization function:

(8) $$\text{Max}LT(x)=\min(\text{max}(EnC_{\text{Pkt}}(x)))x\in V$$

The equation indicates that maximizing network lifetime is a function of minimizing the energy depletion rate of sensor nodes with high energy consumption. Consequently, we proposed a scheme that prevents the quick depletion of the sensor nodes with the highest consumed energy, by balancing the energy usage inside the WSN to route the packets in an efficient way.

### 3.4. The Traffic Generation Model

Applications and their traffic features in sensor networks differ from conventional networks; consequently, packet generation is different. Presently, four traffic types are frequently used in sensor networks: event based, consecutive, query based and hybrid generation. The traffic model has a major impact on the design of the protocol and affects its execution. This paper will focus on the first type, event based generation, which is very popular in WSNs. In this type, the nodes monitor the happening events constantly; if an event arises, the sensor starts to broadcast the event with its value to the collector node. Therefore, within this operation, the routing protocol should be started to find the route to the target node. Based on the occurrence of the events, our on demand routing protocol will take the suitable and efficient actions for these traffic generations.

### 3.5. The Sensor Node Model

**Figure 2.**
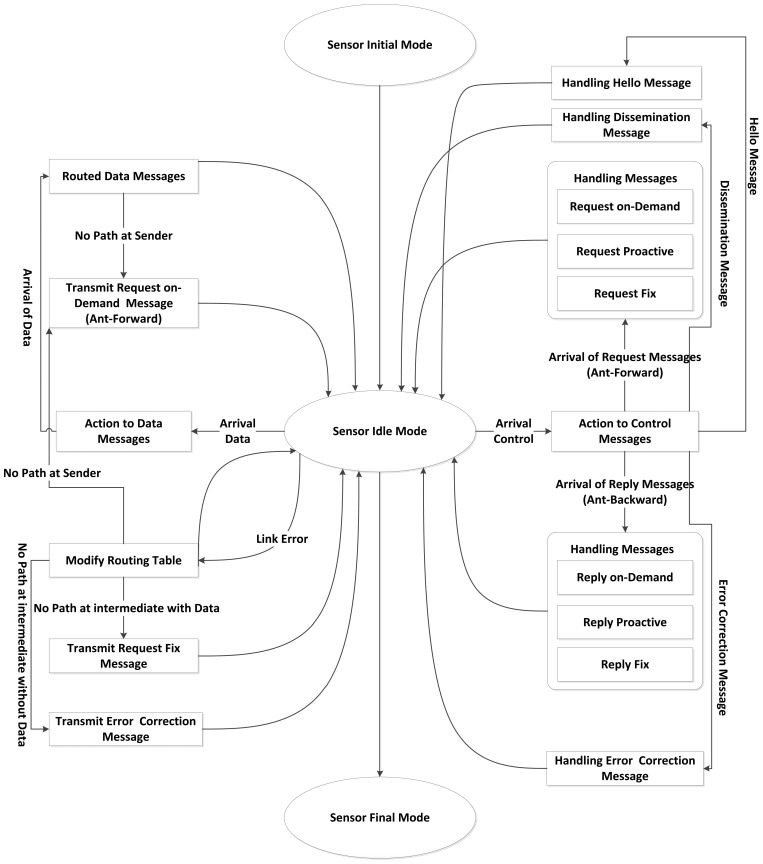
The sensor node model.

In this paper, we modeled the behavior of the sensor nodes in three modes: initial, idle and final as explained in Figure 2. In the initial mode, the algorithm starts by initializing the parameters and then comes to the idle mode where the response to the events occurs. The occurred events are of three types: the receipt of data, control and link error messages.

If the event is a received data message, the scheme starts to route the packets to the sink node. If the current sensor is the sender of the data message, it checks the existence of the path if no information it sends the request on-demand (Ant-Forward) message.

If the received event is a control message either on-demand, proactive or fixed, the protocol verifies this kind of message in order to take suitable action. If it is the request (Ant-Forward) or reply (Ant-Backward) messages, the sensor handles it with the appropriate forward or backward method, from the sender towards the sink and *vice versa*, in order to adjust its routing table accordingly. In case the control message is a hello or dissemination pheromone packet, the sensor needs to get notices that still have a connection with the current sender neighbor; consequently, it derives the information inside the message to modify its routing table.

If the received event is a link error message, the sensor starts to change its routing table to reveal the modified status. Later, if the sink becomes inaccessible, due to link error, the sensor is required to transmit a suitable response; if the sender node lost the path to sink, it will automatically transmits the request on-demand message. On the contrary, if the sensor is an intermediate node, it checks whether the error is due to failure to transmit a data message, if this is the case, it begins to transmit a request fixed message. Otherwise, if the error due to failing to transmit a control message, it sends an error message correction.

### 3.6. SensorAnt Description

**Figure 3.**
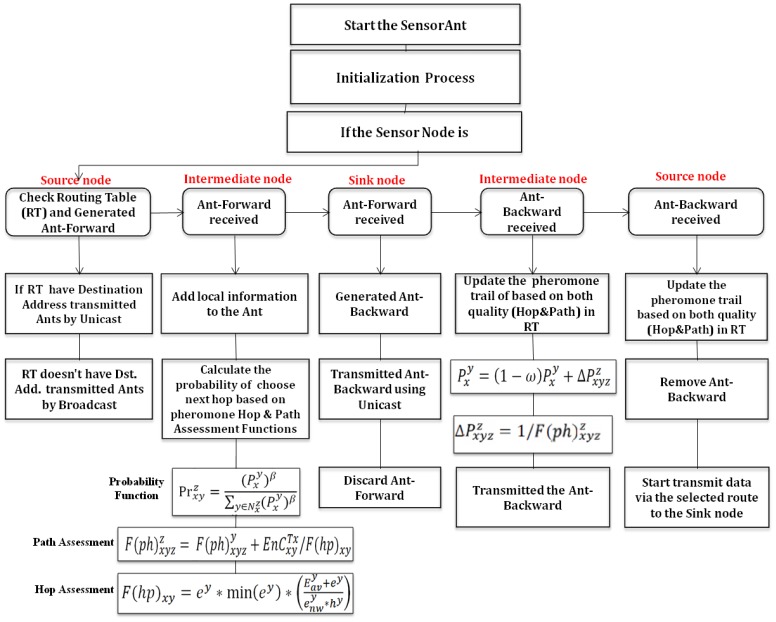
The framework of the proposed protocol.

In our method, SensorAnt will use both on-demand and proactive mechanisms, where the on-demand process will trigger when the sensor node has a data packet to be sent to the sink whereas no information is available in its routing table. In contrast, the proactive starts during the communication period to update the information of WSN and use it later during route maintenance steps. In addition, this operation provides multiple choices of routes to respond with any malfunctioning in WSN during the failure links or fast run-out of sensor's battery. In both mechanisms we apply the route optimizing concept resulting from the ant agent system. The data routing will be updated dynamically during the reinforcement learning process and the failure will be locally fixed. The framework of the proposed SensorAnt protocol is shown in Figure 3. The operations' details of the proposed protocol are described in the following subsections.

#### 3.6.1. Ant Types

**Figure 4.**
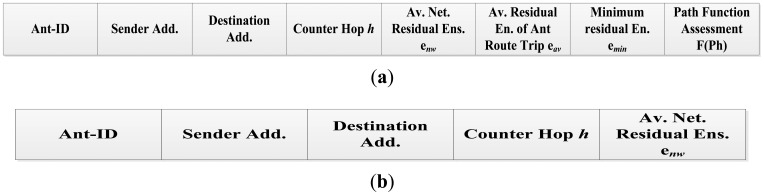
Messages scheme (**a**) request Ant-Forward; (**b**) reply Ant-Backward.

Two types of ants are used during the path discovery and maintenance operations; request Ant-Forward (Ant-F) and reply Ant-Backward (Ant-B). Both ants share some fields of their packet structure such as Ant-ID, originator's source address, the destination address, the counter hop *h* and the total average energy of the sensor network e*_nw_*. Additionally the Ant-F has these fields: the average of the residual energy of the traveling ant route e*_av_*, the minimum residual sensor energy of the sensors during the ant trip e*_min_* and the function quality of the ant route which is updated at each visited sensor *F*(*ph*). Figure 4(a,b) explain the messages scheme of the Ant-Forward and Ant-Backward, respectively.

#### 3.6.2. Pheromone Tables

In our protocol, each sensor node *x* maintains a pheromone table (routing table) *R_x_*. Each entry in the table *R ^z^_xy_* includes the information of the path from sensor *x* to the base station (sink) *z* through its neighbor *y*. The value of both pheromones are referred to as real and non-real, whereby the real pheromone indicates the path quality from sensor *x* to the base station (sink) *z* through its neighbor *y*, gathered by Ant-Forward (Ant-F) and updated by Ant-Backward (Ant-B). The non-real pheromone which indicates the alternative path quality from sensor *x* to the base station (sink) *z* through its neighbor, will be obtained during path recovery stage by using the reinforcement learning mechanism. Therefore, this table keeps the data routing up-to-date using the pheromone trail collected by Ant-F and reinforced by Ant-B, which tracks back the same route constructed by Ant-F.

#### 3.6.3. Path Discovery Steps

When the SN senses data to forward to the base-station (sink), it will check inside its routing table to verify the existing route to the sink. If no data is available, the sender sensor will start to broadcast Ant-F to find out the route to the sink; otherwise, it will send unicast Ant-F using the pheromone information and quality function of both path and hop to select the next hop from *x* to *y* toward sink *z*. It uses the ant agent probability $Pr_{xy}^{z}$ given as follows:

(9) $${\text{Pr}}_{xy}^{z}=\frac{{\left(P_{x}^{y}\right)}^{\beta }}{\sum_{y\in N_{x}^{z}}{\left(P_{x}^{y}\right)}^{\beta }}$$

where $P_{x}^{y}$ is the pheromone trail value of the link (*x,y*) and *β* is the weight factor of the pheromone trail.

During the reception of an Ant-F message from the sensor node *x* to the sink *z* through *y*, it starts assessing the function quality of the ant route, $F{\left(ph\right)}_{\text{xyz}}^{z}$ as follows:

(10) $$F{\left(ph\right)}_{\text{xyz}}^{z}=F{\left(ph\right)}_{\text{xyz}}^{y}+EnC_{xy}^{Tx}/F{\left(hp\right)}_{xy}$$

where $F{\left(ph\right)}_{\text{xyz}}^{y}$ is the quality of the route from the sender of Ant-F at the intermediate sensor *y*, $EnC_{xy}^{Tx}$ is the consumed energy to forward the data from *x* to *y* and *F*(*hp*)*_xy_* is the function to assess the quality between neighbor sensors *x* and *y*, and is given as:

(11) $$F{\left(hp\right)}_{xy}=e^{y}*e_{\text{min}}^{y}*\left(\frac{e_{av}^{y}+e^{y}}{e_{nw}^{y}*h^{y}}\right)$$

where *e^y^* is the current residual energy of sensor *y*, $e_{\text{min}}^{y}$ is minimum residual energy of sensors visited by Ant-F given as $\text{Min}[e_{\text{min}}^{x},e^{y}],e_{av}^{y}$ is the average residual energy of the route from sender *x* until the current sensor *y*, *h^y^* is hop count up to *y* and $e_{nw}^{y}$ is the total average of the whole network residual energies which is computed as the initial energy of the sensor at the first time and later updated with Ant-F and Ant-B using:

(12) $$e_{nw}^{y}=\alpha e_{nw}^{x}+(1-\alpha )e_{nw}^{y}$$

where *α* is the parameter for updating the ratio of predictable and received network average energy.

Consequently, based on the hop function *F*(*hp*)*_xy_*, our model satisfies the balancing of energy consumption that reduces sensor's energy usage in the path and prolong the network life span. This is done by preventing the frequent usage of sensor nodes based on a minimum energy level used here. Hence, the sensor nodes with maximum residual energy will take the role of transmitting the data. Moreover, the paths with high average residual energy will get more opportunities to route the data to the sink.

In addition, the results of hop quality function will be applied to Equation (10) to measure the whole ant tour route from the sender sensor node to the sink. In this case the higher value associated with this route function will be given less path average, which is the better path to be chosen. As a result, the protocol will use this to update the pheromone trail $P_{x}^{y}$ at the pheromone table as follows:

(13) $$P_{x}^{y}=(1-\omega )P_{x}^{y}+\Delta P_{\text{xyz}}^{z}$$

where *ω* is the parameter used to control the decay of the pheromone during the searching process since the last update and $\Delta P_{\text{xyz}}^{z}$ is the increasing value of the ant deposit trail during the journey from the sender node to the sink. This is given as the inverse of function quality of the route from source sensor to the sink:

(14) $$\Delta P_{\text{xyz}}^{z}=1/F{\left(ph\right)}_{\text{xyz}}^{z}$$

Therefore, the route with better quality that has both highest value of minimum residual energy and average route energy will get the higher pheromone trail. In this way the route with best path quality will reinforce and get a better chance to be chosen.

Up to this point each sensor may receive multiple copies of the same Ant-F therefore it removes all duplicate copies that arrive after the first Ant-F. Additionally, the sensor looks for the sink address carried by the Ant-F; if it is different from the current one it will broadcast the Ant-F and follow the same procedure as before, otherwise, it will convert the Ant-F to Ant-B.

When the sink node receives the Ant-F it will generate Ant-B to begin the route reply by using the unicast operation using the same path used by the Ant-F forwarded to the sender node. During the route reply stage using Ant-B, each sensor that receives the ant updates its routing table using Equation (13). When Ant-B reaches the final target sensor -*the sensor generates Ant-F*- the process of searching path finished and the ant is removed. Hence, by applying this technique for several iterations the sensor will be able to find the optimal route to transmit the data to the sink.

#### 3.6.4. Path Recovery Steps

**Figure 5.**
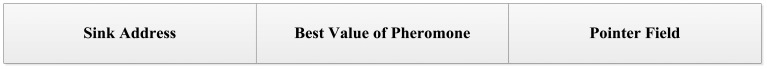
The dissemination message scheme.

In this proposed method, when the source transmits data to the sink within a period of this session it will start sending Ant-F to explore and update the information about the current routes used and check for any other better routes using a proactive mechanism. This process periodically checks the changes in the WSN caused by link failured, either by movement of the sensor nodes or the depletion of sensor nodes in the available path. This process involves the same concept of path searching. It updates the pheromone information to guide the ants to track new routes for data transmission by spreading the pheromone value using the dissemination message, to its neighbors and checking the energy level to compute path quality function of alternative routes. Figure 5 described the structure of the dissemination message where the pointer field indicates if the pheromone value is real or non-real. The non-real pheromone, is obtained by reinforcement learning scheme to update the pheromone table, replacing the maximum (best) value of the pheromone trace based on the multi-criteria function qualities, of both path and hop, which frequently monitor the energy level of the sensors in relaying the data. The advantages is that, this strategy will avoid routing the data packet using the same path all the time and prevent the sensor node from being drained very quickly, resulting in prolonged network life time. This mechanism hence increases the chances of selecting a better route between multiple paths available to forward packets to the sink.

## 4. Performance Evaluation

The results of several simulation experiments are examined and evaluated in order to show the performance under different WSN conditions. The aim of the experiments is to assess the ability and test the effectiveness of the proposed algorithm.

### 4.1. Simulation Environment

**Figure 6.**
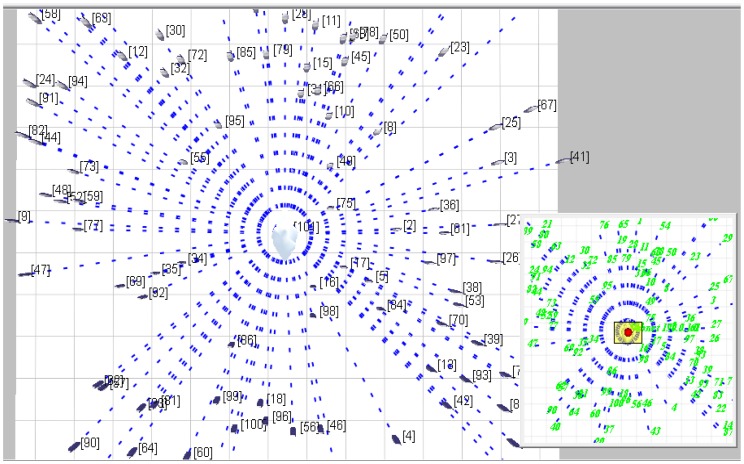
The network model for 100 sensor nodes in flat topology.

The proposed SensorAnt scheme is simulated by deploying different sensor nodes from 10 to 100 based on five different fields to reflect different sensor nodes density—200 × 200 m^2^ for 10 sensor nodes, 300 × 300 m^2^ for 20 sensor nodes, 400 × 400 m^2^ for 30 sensor nodes, 500 × 500 m^2^ for 40 sensor nodes and 600 × 600 m^2^ for 50, 60, 70, 80, 90 and 100 respectively. Figure 6 describes the network model which is flat topology based for one scenario used in this paper. The figure represents the snapshots from the QualNet it contains 100 sensor nodes deployed randomly in the field area 600 × 600 m^2^. All the sensor nodes have the same characteristics and operated with identical battery power. The blue links in the figure indicate to the wireless link that connected all the nodes to subnet (network field). While the green links indicate to the messages exchange during the simulation run time inside the sensor network, and the green numbers indicate to the sensor identification (sensor-ID).

The other parameters used in our simulation are listed in the Table 1. The results of SensorAnt scheme are compared with Energy Efficient Ant-Based Routing (EEABR). In addition, to be more objective we have re-implemented the EEABR routing protocol to work in QualNet libraries in order to run both methods under the same simulator with similar software and hardware platforms. We then tested and validated our SensorAnt method under the same scenarios and simulation setting used by EEABR, in order to prove the effectiveness of our method.

### 4.2. Performance Metrics Evaluation

The main goal of the proposed protocol is to improve the system performance by reducing the energy consumption and extending network lifetime. Following are the metrics that used for evaluation in this paper:

1. **Energy Efficiency**: This term can be declared as the ratio between total consumed energy over the number of packets received by the sink-node.
2. **Energy consumption**: The metric gives the energy consumption of nodes in the event area for transmitting a data packet to sink.
3. **Average Energy**: The metric gives the average of energy of all nodes at the end of simulation.
4. **The standard deviation of energy**: The metric gives the average variance between energy levels on all nodes.
5. **Throughput**: This metric is defined as the rate of the successful packets that can be transferred from a source to the sink node within a certain period of time.

### 4.3. Experimental Results and Discussion

The results and discussion were presented in this section to validate the effectiveness of the SensorAnt comparing with EEABR results; the results obtained from the QualNet simulator that offers high fidelity simulations for wireless communication. The result was studied based on network size, mobility and under different periods of times to show the accuracy of the proposed protocol.

#### 4.3.1. The Impact of Network Size

**Figure 7.**
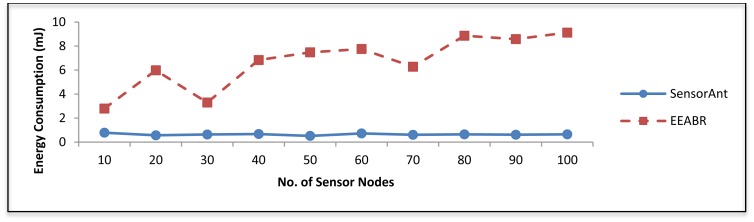
Total energy consumption for different WSNs with different sensor nodes.

Several experiments results have been conducted to verify the impact of the network size. Figure 7 shows the comparison of the proposed SensorAnt with the EEABR model in terms of total energy consumption. Here, our SensorAnt shows better performance in terms of energy conservation compared to the EEABR algorithm. As can be seen, the total energy consumption for SensorAnt are smaller than EEABR with different SNs. This is because SensorAnt can optimize the energy usage of SNs inside WSN and explore the better path based on both function qualities used in our model. The SNs in SensorAnt always monitor their energy level to avoid those SNs with least energy from being used for an extended period of time. Clearly, EEABR exhausted much more energy at large number of sensor nodes. These prove that SensorAnt is an efficient mechanism to decrease the energy usage.

**Figure 8.**
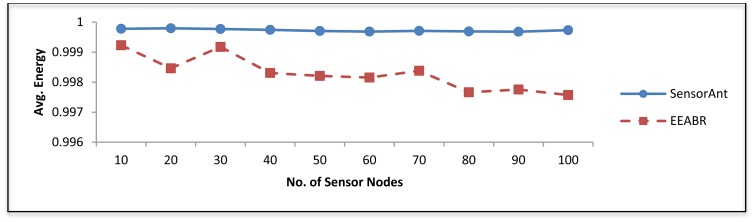
Average energy for different WSNs with different sensor nodes.

Figure 8 illustrates the average energy of all sensor nodes at the end of the simulation. Clearly, energy depletion rate in SensorAnt is fairly constant over the various nodes density. The reason is that SensorAnt employs balanced scheme and equalizes the energy of sensor nodes throughout the system life time.

**Figure 9.**
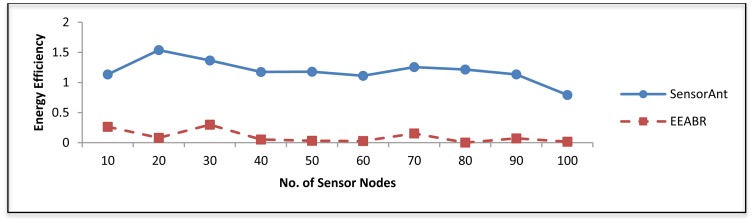
Energy efficiency for different WSNs with different sensor nodes.

Figure 9 shows the performance of energy efficiency in SensorAnt compared with EEABR; which is defined as the ratio between the whole energy consumption and the total of messages reached at the sink. It can be observed that the energy efficiency of our SensorAnt far outperforms EEABR; this is because SensorAnt receives more packets with the lowest energy-cost than EEABR. This stems from the fact that our model minimizes the number of dropped packets by preventing congestions hence this limits the number of retransmissions.

**Figure 10.**
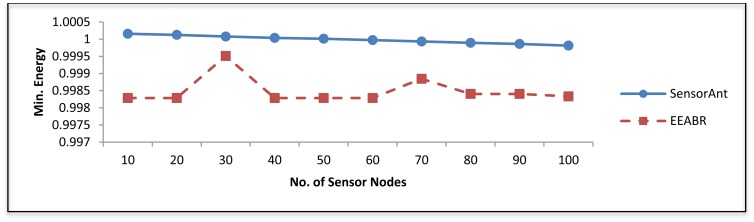
Minimum energy for different WSNs with different sensor nodes.

Figure 10 shows the lowest energy among all sensor nodes. As can be seen our SensorAnt gives the best result compare with EEABR; this is because in WSN most of the nodes play the role as routers, and data forwarding which consumes much energy, whereas our SensorAnt uses multiple alternative routes that have best qualities.

**Figure 11.**
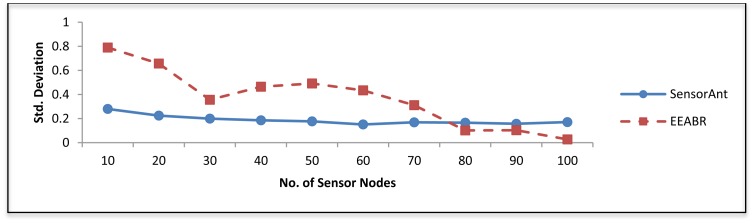
Standard deviation for different WSNs with different sensor nodes.

Figure 11 shows the standard deviation of SensorAnt and EEABR. As can be seen SensorAnt gave the smallest changes; this is because it utilizes the sensor nodes most fairly. This implies that, if the number of communications increases, the energy of sensor nod's inside the WSN can be balanced better.

**Figure 12.**
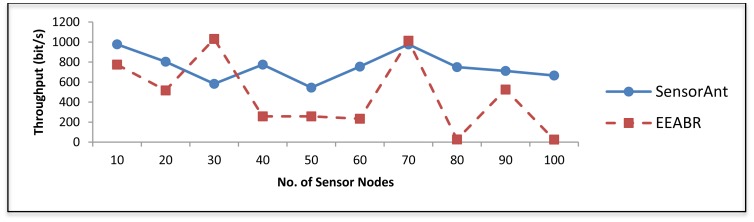
Throughput for different WSNs with different sensor nodes.

As can be seen in Figure 12 our SensorAnt performs much better than EEABR in terms of throughput; this is because our model prevents congestion when the traffic load becomes heavier by distributing it over multiple routes. This is done in our proposed method using a proactive mechanism during the period of data transmission from the sender to the sink which periodically checks the energy status of WSN and selects the path with a better function quality by assigning more pheromone to it through positive reinforcement while the paths with little energy will get negative reinforcement.

**Figure 13.**
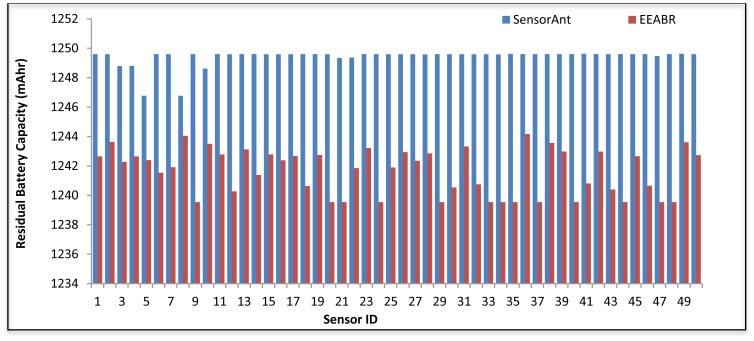
Energy balanced of SensorAnt and EEABR for 50 sensor nodes at 2,000 s.

**Figure 14.**
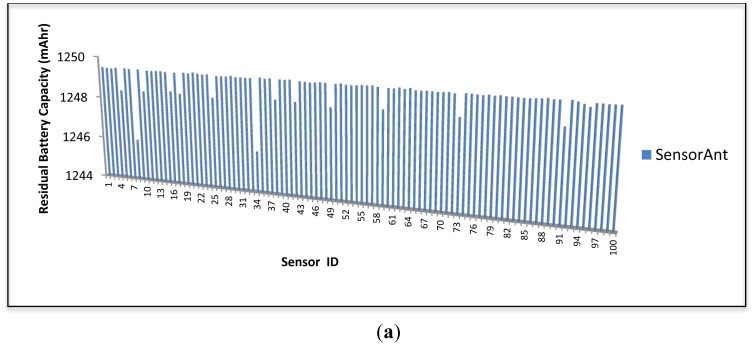

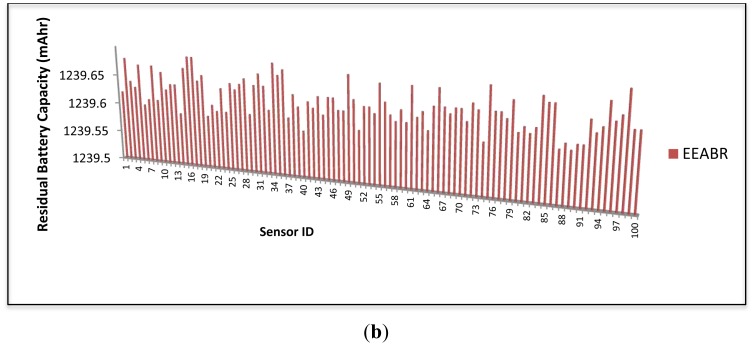
Energy balanced with 100 Sensor nodes at 2,000 s for (**a**) SensorAnt and (**b**) EEABR.

Figure 13 and Figure 14(a,b) show the energy balance plots of both algorithms. As can be seen, SensorAnt attains the more preferred energy balance in both the 50 and 100 sensor networks compared to the EEABR scheme over the 2,000 s period. Whereas the sensors in EEABR drained their battery energy rapidly, almost all sensor nodes in SensorAnt still have a high battery capacity which allows them to live longer and keep responding to the occurring events. This phenomenon is due to the fact that the packet traffic of EEABR scheme focuses on particular nodes that are close to the sink or located in the efficient route as outlined by its proactive method. In contrast, SensorAnt distributes the energy consumption over the whole WSN thereby achieving the required energy balance across the network. Accordingly, the sensor network life span is extended.

**Figure 15.**
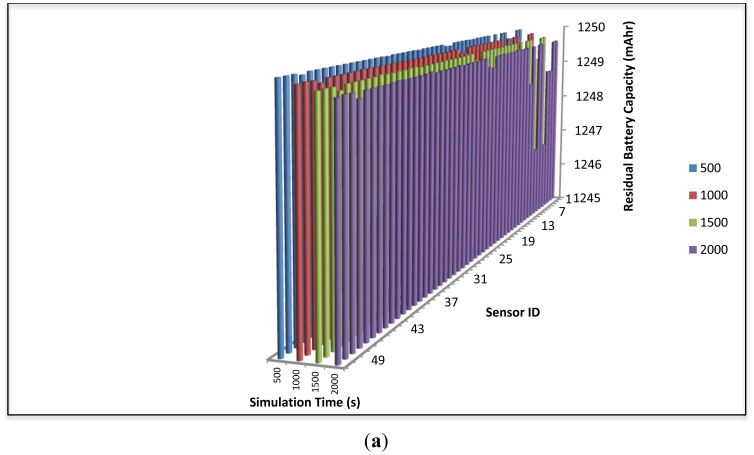

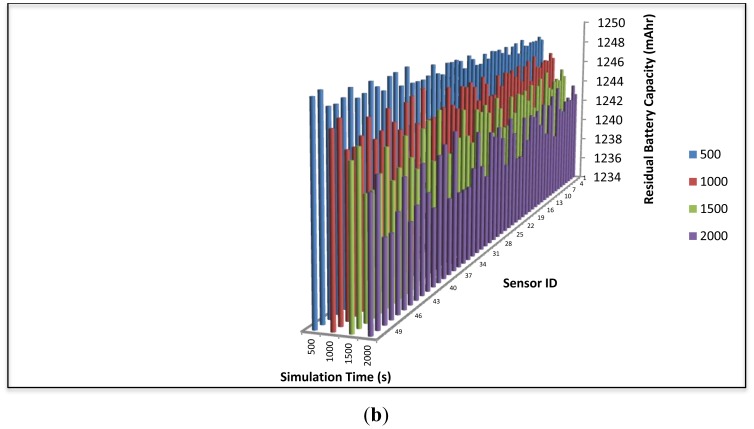
Energy balanced for 50 sensors with different times for (**a**) SensorAnt and (**b**) EEABR.

Figure 15(a,b) illustrate the residual battery capacity of SensorAnt and EEABR respectively, after different periods of time. In Figure 15(a), it is observed that in the proposed SensorAnt, the energy residue is still balanced across the sensor nodes even if the battery depleted slowly and regularly with increasing run time. This is due to the self-adaptive scheme employed in the proposed SensorAnt which always monitors the sensors' remaining energies and only permits those sensor nodes with high residual energy to participate in the routing function. On the other hand, Figure 15(b) shows clearly that the sensor nodes in the EEABR scheme exhaust their residual energy very fast as the run time increases; which is due to the lack of an appropriate energy consumption distribution among the sensor nodes. Instead, the scheme only focuses on maintaining the efficient route at the expense of increased energy depletion.

As a result, while SensorAnt attempts to maximize the uniformity; the traffic is scattered through the whole WSN leading to better energy balancing. This adequate balancing comes from the efficient way of exploiting the battery resources.

**Figure 16.**
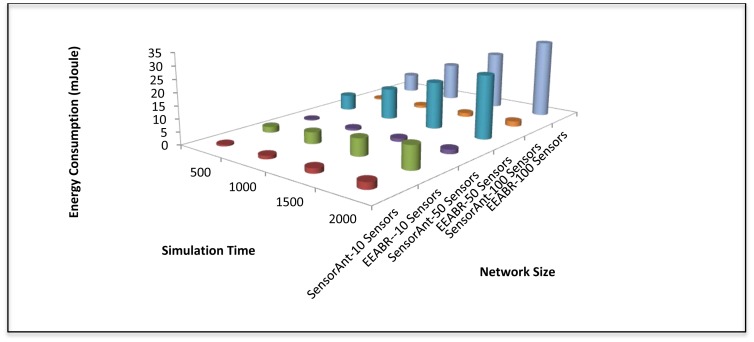
Total energy consumption for different networks under different time for SensorAnt and EEABR.

Figure 16 shows the total energy consumption of the two schemes with time. Again, it can be clearly seen that the proposed SensorAnt presents a superior performance compared to the EEABR. Here, the energy consumption increases gradually with time when the proposed SensorAnt is employed. However, with the EEABR scheme, energy consumption increases rapidly with the time and sensor nodes increase.

In these experiments, we observe that our SensorAnt shows better energy efficiency than EEABR. Increased number of nodes EEABR consumes energy faster, whereas SensorAnt proceeds to become more stable. This is because in our model, Ant-B find out paths to destination according to the pheromone trail connected with the current WSN energy state gathered by Ant-F. After a period of transmission, the remaining energy of sensor nodes are changed for different traffic, accordingly the WSN status will be affected, then a different route with better function quality will be selected to assist the following transmission during the path search stage. Additionally, when the traffic load from the sender to destination stays for some length of time and becomes heavier, sensor's residual energy in SensorAnt will be checked occasionally and if it found that the energy drains fast a new path searching operation will be started and new paths will be established that avoids over use of affected SNs.

#### 4.3.2. The Impact of Sensor Mobility

In this section, several experiments have been conducted to verify the impact of sensor nodes mobility on the performance of the proposed SensorAnt. To achieve this, the Static-SensorAnt deployment is compared with another SensorAnt scenario where all the nodes in the network are mobile using the random way point mobility model. The effects of this operation are studied from two prospective: first, under various numbers of sensor nodes and second under different time periods using same performance metrics as before.

##### (a) Under Varying Number of Sensor Nodes

**Figure 17.**
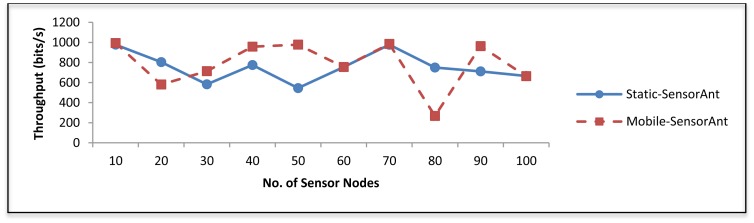
Comparison of throughput between Static and Mobile-SensorAnt with different sensors.

the result presented here compares the Static-SensorAnt and the Mobile-SensorAnt as the number of sensor nodes varies. Figure 17 demonstrates that the Mobile-SensorAnt obtains better throughput compared to the Static-SensorAnt. This is because in the static case, the congestion and message drops occur more than in the mobile case. Moreover, notice that the difference between the two cases is not too much, which is indicative of the fact that adaptation of the proposed SensorAnt scheme from the static to a mobile environment is indeed feasible.

**Figure 18.**
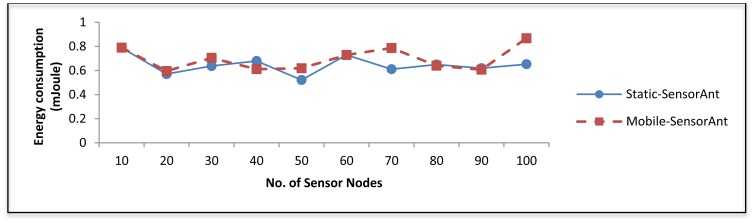
Comparison of total energy consumption for Static and Mobile-SensorAnt with different Sensor Nodes.

Also, Figure 18 shows that the Static-SensorAnt presents a better performance than the Mobile-SensorAnt with regards to the total energy consumption, which is observably comparable. This phenomenon can be attributed to the fact that the mobile nodes which act as relays are regularly changed leading to a much fairer distribution of the consumed energy among the sensor nodes. It is significant to observe that the energy usage is associated with the throughput whereas packet drops increase the consumed energy.

**Figure 19.**
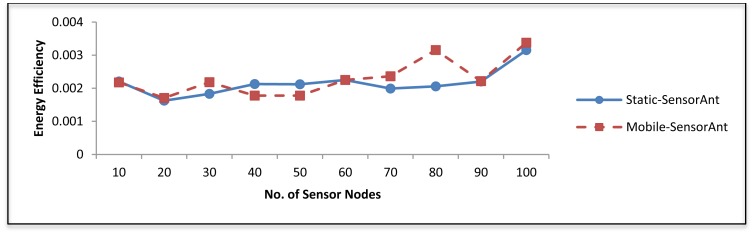
Comparison of energy efficiency between Static and Mobile-SensorAnt with different sensors.

**Figure 20.**
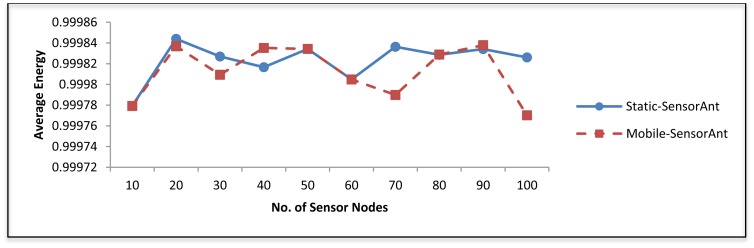
Comparison of average energy for Static and Mobile-SensorAnt with different sensors.

**Figure 21.**
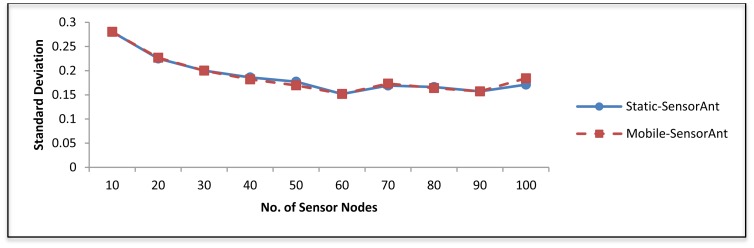
Comparison of standard deviation between Static and Mobile-SensorAnt with different Sensor Nodes.

Figures 19 and 20 depict the energy efficiency and average energy of the deployed sensors for both the static and mobile SensorAnt. As it can be seen, the Mobile-SensorAnt incurs a little more energy cost compared to the Static-SensorAnt but is still acceptable. Increasing the network size under the same period of time doesn't make sense, since we applied the energy-balance scheme, in both situations, where the energy consumption is totally balanced between nodes. Figure 21 clarifies this further, showing the standard deviation of the energy cost for both scenarios. As in the previous plots, the deviations in energy levels between the static and mobile scenarios are very similar; thus validating the effectiveness of the SensorAnt algorithm.

##### (b) Under Different Periods of Time

**Figure 22.**
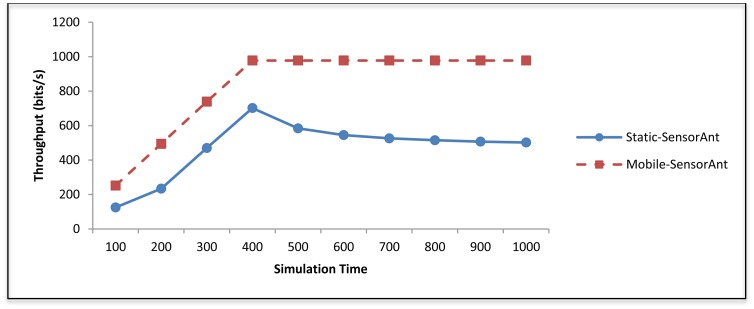
Comparison of throughput between Static and Mobile-SensorAnt with different periods of time.

The result presented here compares the energy cost incurred by the Static and Mobile-SensorAnt scenarios with time. Figure 22 shows that the Mobile-SensorAnt outperforms the Static-SensorAnt throughout the simulation time. In the static case, the congestion and packet drops occur more than in the mobile case. It can be noted that in both cases, the first 400 s of the simulation time depicts increasing throughput; however, beyond this point, Mobile-SensorAnt stabilizes whereas the Static-SensorAnt shows decreasing trends in throughput. This is so because as the path between the sender and sink nodes becomes congested with more packets, the occurrence of packet drops is likely to increase when the network is not dynamic. In the Mobile-SensorAnt however, the sensor nodes keep moving about in the WSN, thereby resulting in the re-distribution of the system bottlenecks throughput the whole WSN.

**Figure 23.**
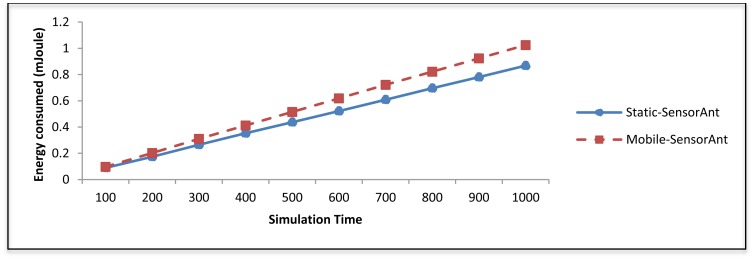
Total energy consumption between Static and Mobile-SensorAnt with different periods of time.

Figure 23 illustrates the total energy usage of the proposed Static-SensorAnt compared to the Mobile-SensorAnt. Here, it can be clearly seen that the Static-SensorAnt decreases the energy usage compared to the Mobile-SensorAnt under all circumstances. By investigating the throughput and the energy consumption of the Mobile-SensorAnt, we observed that the Mobile-SensorAnt got a significant packets' delivery resulting to cost higher energy than Static-SensorAnt. In addition, we can see from the same figure that with increasing the time the consumption of the energy increases too for both cases due to the packet's transmission.

**Figure 24.**
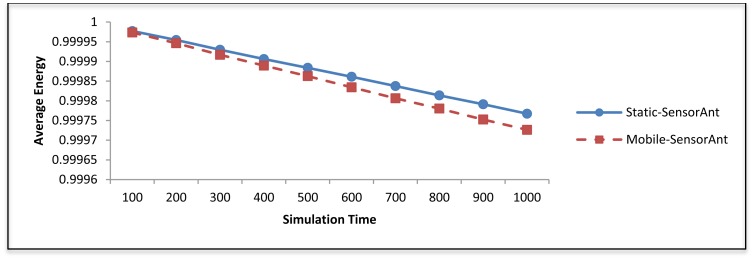
Average energy of Static and Mobile-SensorAnt with different periods of time.

**Figure 25.**
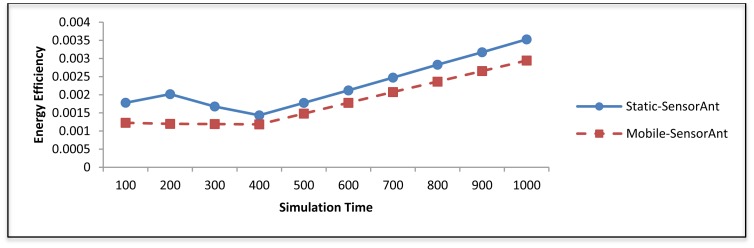
The energy efficiency of the Static and Mobile-SensorAnt with different periods of time.

In Figure 24, the average remaining energy of the sensor nodes in both the Static and the Mobile-SensorAnt are shown. Here, the static case shows better results compared to the mobile case. This is because in the mobile case, most of the sensor nodes move away from their current routes with time, which imposes more computational complexity on the algorithm in order to discover new paths, resulting in the dissipation of more energy; thereby resulting in the exhaustion of the average residual energy in the WSN. This is in fact affects the energy efficiency of the Mobile-SensorAnt as shown in Figure 25 where energy efficiency of Static-SensorAnt outperforms Mobile-SensorAnt with increasing simulation time.

**Figure 26.**
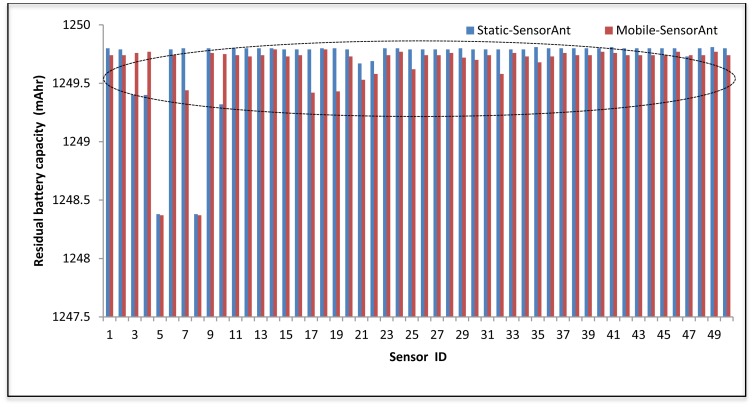
The energy balancing of the Static and Mobile-SensorAnt at time 1,000 s with 50 sensors.

From Figure 26, it can be seen that the energy balanced at the 1,000 s of time in the static scenario compared with the mobile in our proposed method, nevertheless the result still comparable. This is because the route selection in the static case is more stable by causing the distribution of the available energy among the neighboring sensor nodes with fixed locations. In this way, the energy consumption is fairly balanced over some sensors in the WSN compared to the Mobile-SensorAnt.

## 5. Conclusions

In this paper we described a method to optimize energy consumption in a wireless sensor network, considering that the energy of the sensor nodes is the most critical factor to prevent fast energy depletion. Inspired by the colony of ants, we presented SensorAnt to use a new routing scheme to optimize the battery power of sensor nodes participating in the paths to forward the data across the sensor networks. Hence, we introduced two function qualities for the hop and path to improve the selection of the best routes available. Then, we discussed the obtained experimental results and the validity of the proposed SensorAnt scheme. The simulation results and the observations of several experiments showed that the proposed SensorAnt has superior performance compared to EEABR in terms of total energy usage, energy efficiency and energy balancing.

Investigation into the energy overhead of the proposed routing algorithm, especially during the route decision making needs to be computed in order to ascertain the impact of this wastage of energy on the multiple hop routing model. Therefore, further works will focus on finding out whether there is any considerable effect of overhead energy on the network life. Moreover, this work may also be extended in order to analyze other Quality of Service (QoS) metrics such as delay and average jitter. This is useful to assess if there is any tradeoff between QoS and energy awareness metrics in the proposed decentralized scheme.

## Figures and Tables

**Table 1.** Simulation setting.

**Name**	**Value**
Simulation Tool	QualNet V5
Channel Frequency	2.4 GHz
Traffic	CBR
Packet Size	32 byte
Interval Time	1 second
Energy Model	Micaz
Data Rate	250 kbps
Initial Energy	1,250 mJ

## References

[1] Yick J., Mukherjee B., Ghosal D. (2008). Wireless sensor network survey. Comput. Netw..

[2] Baronti P., Pillai P., Chook V.W.C., Chessa S., Gotta A., Hu Y.F. (2007). Wireless sensor networks: A survey on the state of the art and the 802.15.4 and ZigBee standards. Comput. Commun..

[3] Anastasi G., Conti M., Di Francesco M., Passarella A. (2009). Energy conservation in wireless sensor networks: A survey. Ad Hoc Netw..

[4] Kandris D., Tsioumas P., Tzes A., Nikolakopoulos G., Vergados D.D. (2009). Power conservation through energy efficient routing in wireless sensor networks. Sensors.

[5] Al-Karaki J.N., Kamal A.E. (2004). Routing techniques in wireless sensor networks: A survey. Wirel. Commun. IEEE..

[6] Saleem M., Di Caro G.A., Farooq M. (2011). Swarm intelligence based routing protocol for wireless sensor networks: Survey and future directions. Inf. Sci..

[7] Dorigo M., Stützle T. (2004). Ant Colony Optimization.

[8] Camilo T., Carreto C., Silva J., Boavida F. An Energy-Efficient Ant-Based Routing Algorithm for Wireless Sensor Networks.

[9] Wang X., Ma J.-J., Wang S., Bi D.-W. (2007). Cluster-based dynamic energy management for collaborative target tracking in wireless sensor networks. Sensors.

[10] El-Hoiydi A., Decotignie J. (2004). WiseMAC: An ultra low power MAC protocol for multi-hop wireless sensor networks. Algorithmic Asp. Wirel. Sens. Netw..

[11] Chen M., Kwon T., Mao S., Yuan Y. (2008). Reliable and energy-efficient routing protocol in dense wireless sensor networks. Int. J. Sens. Netw. IJSNET.

[12] Jae-Hwan C., Tassiulas L. (2004). Maximum lifetime routing in wireless sensor networks. IEEE ACM Trans. Netw..

[13] Bonabeau E., Dorigo M., Theraulaz G. (1999). Swarm Intelligence: From Natural to Artificial Systems.

[14] Dorigo M., Caro G.D., Gambardella L.M. (1999). Ant algorithms for discrete optimization. Artif. Life.

[15] Singh G., Das S., Gosavi S.V., Pujar S., Sugumaran V. (2008). Ant Colony Algorithms for Steiner Trees: An Application to Routing In Sensor Networks. Intelligent Information Technologies: Concepts, Methodologies, Tools, and Applications.

[16] Ghasem A.R., Rahman A.M., Rahman M.A., Gueaieb W. Ant Colony-Based Many-to-One Sensory Data Routing in Wireless Sensor Networks.

[17] Misra R., Mandal C. Ant-Aggregation: Ant Colony Algorithm for optimal Data Aggregation in Wireless Sensor Networks.

[18] Salehpour A.A., Mirmobin B., Afzali-Kusha A., Mohammadi S. An Energy Efficient Routing Protocol for Cluster-Based Wireless Sensor Networks Using Ant Colony Optimization.

[19] Heinzelman W.R., Chandrakasan A., Balakrishnan H. Energy-Efficient Communication Protocol for Wireless Microsensor Networks.

[20] Matrouk K., Landfeldt B. (2009). RETT-gen: A globally efficient routing protocol for wireless sensor networks by equalising sensor energy and avoiding energy holes. Ad Hoc Netw..

[21] Lindsey S., Raghavendra C.S. PEGASIS: Power-Efficient Gathering in Sensor Information Systems.

[22] Manjeshwar A., Agrawal D.P. TEEN: A Routing Protocol for enhanced Efficiency in Wireless Sensor Networks.

[23] Rogers A., David E., Jennings N.R. (2005). Self-organized routing for wireless microsensor networks. IEEE Trans. Sys. Man. Cybern. A..

[24] Intanagonwiwat C., Govindan R., Estrin D. Directed Diffusion: A Scalable and Robust Communication Paradigm for Sensor Networks.

[25] Lin M., Marzullo K., Masini S. (1999). Gossip versus Deterministic Flooding: Low Message Overhead and High Reliability for Broadcasting on Small Networks.

[26] Ok C.-S., Lee S., Mitra P., Kumara S. (2009). Distributed energy balanced routing for wireless sensor networks. Comput. Ind. Eng..

[27] Sohraby K., Minoli D., Znati T.F. (2007). Wireless Sensor Networks: Technology, Protocols, and Applications.

